# Snake Venoms in Cancer Therapy: Past, Present and Future

**DOI:** 10.3390/toxins10090346

**Published:** 2018-08-29

**Authors:** Li Li, Jianzhong Huang, Yao Lin

**Affiliations:** 1 Engineering Research Center of Industrial Microbiology, College of Life Sciences, Fujian Normal University, Fuzhou 350117, China; lili@fjnu.edu.cn; 2 Provincial University Key Laboratory of Cellular Stress Response and Metabolic Regulation, College of Life Sciences, Fujian Normal University, Fuzhou 350117, China

**Keywords:** snake venom, cancer, target therapy

## Abstract

Cancer is one of the leading causes of morbidity and mortality worldwide, and the discovery of new drugs for cancer therapy is one of the most important objectives for the pharmaceutical industry. Snake venoms are complex mixtures containing different peptides, proteins, enzymes, carbohydrates and other bioactive molecules, which are secreted by the snake in the predation or defending against threats. Understanding the snake venoms may turn the toxins into a valuable source of new lead compounds in drug discovery. Captopril, the first angiotensin-converting enzyme inhibitor approved in 1981 by FDA, was designed based on the structure of a peptide isolated from the snake venom. The earliest reports about snake venoms used in cancer treatments appeared in the 1930s. Since then, numerous studies on the activities, isolations, purifications and structure elucidations of the components from snake venoms were published. The comprehensive structural and functional investigations of snake venoms would contribute to the development of novel anti-cancer drugs. Our review will focus on the past, present and the future of the studies on snake venoms in cancer target therapy.

## 1. Introduction

Cancer is one of the leading causes of morbidity and mortality worldwide. According to GLOBOCAN, there were approximately 14.1 million new cases diagnosed and 8.2 million deaths from cancer in 2012 globally [1]. Surgery and chemotherapy are still the main strategies for cancer therapy [2]. Target therapy, which interferes with a specific molecular target and usually causes fewer toxicities, is becoming more and more popular in chemotherapy [3,4]. Recently, the compounds purified and characterized from snake venom displayed a tremendous potential as agents targeting specific molecular pathways in cancer cells [5,6].

Snake venoms are complex mixtures of proteins, peptides and other bioactive molecules secreted by the venom gland of snakes and injected by unique fangs of snakes to debilitate and digest their prey. World Health Organization has placed snakebite envenoming on its list of top 20 priority neglected tropical diseases, which kills more than 100,000 people and maims 400,000 people annually [7]. The various clinical manifestations of snakebite victims are caused by the highly complex and diverse compositions of snake venoms, which can selectively recognize their different biological targets [8]. Although snakebite envenoming is a life-threatening public health problem, snake venoms are recognized as a potential resource of biologically active compounds. In China, snake wine or snake venom liquor is supplied as traditional Chinese medicine [9]. Snake venoms are also discovered and developed as drug leads in the modern drug industry. For example, captopril was the first angiotensin-converting enzyme inhibitor approved in 1981 by FDA for the treatment of hypertension and some types of congestive heart failure. Captopril was actually designed based on BPP_5a_, a bradykinin-potentiating pentapeptide isolated from venoms of *Bothrops jararaca* [10].

Common venom components could be classified as enzyme and non-enzyme components. Enzymatic snake venoms include phospholipase A_2_ (PLA_2_), l-amino acid oxidases (LAAO), metalloproteases (SVMP), serine proteases (SVSP), 5′-nucleotidases, acetylcholinesterases and hyaluronidases. Non-enzymatic components include disintegrins (DIS), three-finger toxins (3FTx), Kunitz peptides, cysteine-rich secretory proteins (CRiSP), C-type lectins (CTL) and natriuretic peptides (NP) [11]. There has been a long history of research on exploring the therapeutic potential of snake venoms for cancer.

## 2. Early-Stage Study on Snake Venoms in Cancer Therapy

The effect of snake venoms was first investigated in the 1930s. For example, Essex et al. treated 15 tumor-bearing white rats with intravenous injections of different doses of venoms from rattlesnakes. However, after 6 successive weeks, cancer progresses between the experimental and control groups were similar [12]. Kurotchkin et al. discovered that cobra venom could destroy cells of the Fujinami rat sarcoma, which seemed to require direct contact between the venom and tumor cells [13]. Ligneris et al. showed that African snake venoms had no effect on the great majority of tumors in humans [14]. Notwithstanding, there were no encouraging results on cancer suppression, the pain relief effects of snake venoms were shown in some cases, whose advantages were long-acting and no morphine dependence [14]. In 1936, Macht reported the experimental and clinical study of cobra venoms as pain-relieving agents. In total, 105 cancer patients were injected with a dose of 2–5 mouse units, which was defined as the quantity of venom solution enough to kill a 22 g white mouse within 18 h after intraperitoneal injection [15]. Among the patients, 30 cases showed definite relief and 38 cases showed marked relief. Only 13.3% of the patients showed doubtful results or no relief [16].

Meanwhile, enzymes of snake venoms attracted attention of investigators for their potent biological significances. In 1938, Jynegar et al. found the activity of cholinesterase in cobra venom [17], and Zeller found that cholinesterase exists in many types of snake venom [18]. The activity of hyaluronidase in snake venom was noted by Duran-Reynals in 1936 [19]. The first nonhydrolytic enzyme, l-amino acid oxidase (LAAO), was reported by Zeller in 1944 [20].

In summary, the studies on the inhibitory effect of crude snake venoms towards tumor cells showed doubtful results at the early stages. The snake venom was used as the mixture and their main clinical effect for cancer therapy was pain relief for the patients with hopelessly malignant tumors.

## 3. Development of Snake Venoms for Cancer Target Therapy

The isolation and characterization of the components from snake venoms began in the 1940s. After that, numerous components including enzymes, non-enzyme proteins and peptides were purified, sequenced and structurally elucidated. l-amino acid oxidase (LAAO) was first isolated and characterized by Singer et al. from moccasin snake venom in the early 1950s [21,22,23,24]. LAAO is a FAD (Flavin Adenine Dinucleotide)—containing an enzyme that converts l-amino acid stereospecific into the corresponding α-keto acid with hydrogen peroxide and ammonia as byproducts [25].

Phospholipase A_2_ (PLA_2_) from *Crotalus adamanteus* was purified and partially characterized by Saito and Hanahan in 1962 [26]. In 1969, Wu and Tinker studied PLA_2_ from *Crotalus atrox* [27]. The PLA_2_ enzyme hydrolyzes glycerophospholipid to form lysophopholipid and fatty acid. Snake venoms often contain multiple types of PLA_2_ isoenzymes, resulting in extra difficulty for purification. For example, eight PLA_2_ (Pa-1G, Pa-3, Pa-5, Pa-9C, Pa-10A, Pa-12A, Pa-12C and Pa-15) have been isolated and sequenced from the venom of Australian king brown snake (*Pseudechis australis*) [28].

The lectin-related proteins in snake venom have been classified into true C-type lectins (containing the CRD domain) and C-type lectin-like proteins (containing the CRD-related non-carbohydrate-binding domains) [29,30]. A slice of C-type lectins or C-type lectin-like proteins were isolated from snake venoms in 1970s. Batroxobin, a lectin from *Bothrops atrox* venom, was isolated by Stocker and Barlow in 1976 [31]. Kirby et al. purified and characterized thrombocytin from *B. atrox* venom in 1979 [32]. It was found that C-type lectin binds to a sugar moiety at the presence of Ca^2+^ and contains the carbohydrate recognition domain (CRD).

Snake venom metalloproteinase (SVMP) is a major component in most viperidvenoms, and one of the import enzymes contributing to the toxicity of snake venom [33]. In 1978, Bjarnason and Tu purified SVMPs from western diamondback rattlesnake (*Crotalus atrox*) venom and showed that the zinc in each of these SVMPs was at approximate 1:1 ratio to the relevant protein. Removal of zinc from SVMPs abolished both proteolytic and hemorrhagic activities of the SVMPs [34].

Disintegrins are a family of integrin inhibitory proteins with low molecular weight, tripeptide sequence arginine-glycine-aspartic acid (RGD), and cysteine-rich peptides isolated from various snake venoms [35]. The integrin binding function usually depends upon the RGD motif. However, some disintegrins lacking this RGD motif can also bind and block integrins. In the late 1980s, Huang et al. purified and determined the primary structure of trigramin, a disintegrin from *Trimeresurus gramineus* snake venom, which kicked off a promising research field of the inhibition of integrin function by snake venom [36,37,38]. The isolation and characterization of components of snake venoms paved the way for cancer targeted therapy in modern medicine. Here, we discuss the army of snake venoms with different mechanisms of actions in cancer therapy (Table 1).

### 3.1. Antiangiogenesis

Human tumor growth is accompanied by neovascularization to provide essential nutrition and oxygen. Angiogenesis supports tumor cell extension and invasion into nearby normal tissue and is required to distant metastasis. Antiangiogenesis is a propitious strategy for cancer targeted therapy. Quite a few angiogenesis inhibitors for the treatment of cancer have been approved by FDA including Bevacizumab (targeting vascular endothelial growth factor, VEGF), Sorafenib (tyrosine kinase inhibitror, TKI), Sunitinib (TKI) et al. [53].

Disintegrins purified from snake venoms showed antiangiogenesis effects. Leucurogin, a disintegrin cloned from *Bothrops leucurus* (white-tailed-jararaca), showed significant anticancer activities against Ehrlich tumor implanted in mice with the administration of 10 μg/day. Antiangiogenesis effect of leucurogin was assessed and confirmed by the sponge implant model in mice [39]. Contortrostatin is a homodimeric peptide isolated from the venom of *Agkistrodon contortrix contortrix*, a subspecies of the southern copperhead snake, and contains a RGD sequence [40]. Contortrostatin showed anti-angiogenic activity against the primary tumor of human breast cancer MDA-MB-435 carried in mice. Obtustatin, a disintegrin isolated from *Vipera lebetina obtusa* venom has no classical RGD sequence [54]. Obtustatin reduced tumor size in the Lewis lung syngeneic mouse model and showed 84% inhibition of angiogenesis activities in the experiments of chick Chorioallantoic Membrane (CAM) assay [41]. Adinbitor is a disintegrin cloned from *Agkistrodon halys brevicaudus stejneger* with 73 amino acid residues including 12 cysteines and a RGD motif. Adinbitor can inhibit bFGF-induced proliferation of ECV304 cells with IC_50_ of 0.89 μΜ. In the Chick CAM angiogenesis assay, adinbitor showed the activities against bFGF-induced angiogenesis both in vivo and in vitro [42,43]. Salmosin was purified from the snake venom of *Agkistrodon halys brevicaudus* in 1998 [55]. Salmosin can prevent the bFGF induced bovine capillary endothelial cell proliferation. Treatment with salmosin significantly suppressed the growth of both the metastatic and solid tumor in mouse xenografts of Lewis lung carcinoma cells, and the tumor specific antiangiogenic activity of salmosin was considered related to the blockade of α_v_β_3_ integrin [44].

### 3.2. Apoptosis Induction

Apoptosis is a process of programmed cell death to delete unnecessary cells in normal tissues and to keep cellular homeostasis. Any critical defect in the apoptotic process may lead to uncontrolled growth of cells and result in cancer [56,57]. A subset of snake venom proteins have demonstrated antitumoral activities by inducing apoptosis. In 1993, Araki et al. found that some hemorrhagic snake venoms induced apoptosis of vascular endothelial cells. However, the active component was unknown [58]. After the report of Araki et al., increasing LAAOs from snake venoms have increasingly been shown to induce apoptosis. Suhr and Kim purified and characterized a LAAO from the venom of *Agkistrodon halys*, and exposure to this LAAO resulted in the apoptosis of cultured L1210 cells [45]. Later, the research of Suhr et al. suggested that the activity of apoptosis induction of LAAO was not solely due to the production of H_2_O_2_ in the reaction of LAAO [46].

AHP-LAAO, a novel snake-venom LAAO, was isolated from *A. halys pallas* venom in 2004. The AHP-LAAO inhibited the proliferation of HeLa cells at 0.5 μg/mL and induced DNA fragmentation and nuclear morphological changes [47]. Samel et al. purified and characterized a homodimer LAAO from the venom of the common viper *Vipera berus berus*. The DNA fragmentation gel pattern indicated that the LAAO from *V. berus berus* induced apoptosis in cultured K562 and HeLa cells, and the inhibition of apoptosis by catalase suggested the role of hydrogen peroxide in the process [48].

Not only LAAO but also some disintegrins showed the activities of apoptosis induction. Thangam et al. purified the disintegrin from the venom of the Indian cobra snake (*Naja naja*), whose anticancer activity was at IC_50_ of 2.5 ± 0.5 μg/mL, 3.5 ± 0.5 μg/mL, and 3 ± 0.5 μg/mL for the MCF-7, A549 and HepG2 cell lines respectively. The DNA fragment analysis and AO/EtBr staining assay suggested that this disintegrin induced the apoptosis of the cancer cell lines [49]. Two metalloprotease/disintegrin family proteins, VAP and VAP2, were purified from the venom of the rattlesnake *Crotalus atrox*. The apoptosis-inducing activities seemed to be specific towards endothelial cells [50,51]. Han et al. characterized stejnitin, a SVMP from the venom of *Trimeresurus stejnegeri*. Stejnitin comprises metalloproteinase and disintegrin, and the DNA fragmentation and flow cytometry analysis suggested stejnitin induces apoptosis in ECV304 cells [52].

## 4. Future Directions

**Figure 1:**
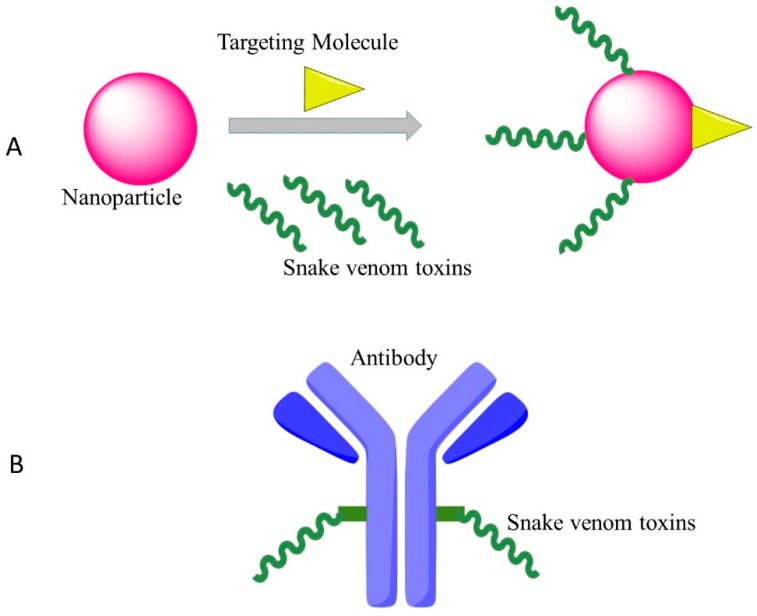
Effective targeting therapy by cytotoxins of snake venoms with new approaches. (**A**): Nanoparticles transport snake venoms to specific locations in the body; (**B**): Snake venoms conjugated with monoclonal antibodies for targeted therapy.

Though there was an ample evidence about the therapeutic potentials of snake venoms in the treatment of cancer, more research is needed. Most components of snake venoms, including PLA_2_s, LAAOs, metalloproteases, disintegrins and other peptides show cytotoxicity to cancer cells. However, the discrimination between normal and cancer cells is the main problem in cancer treatment [59]. From our perspective, future research could pour more attention into these actions (Figure 1).

### 4.1. Isolation and Characterization of New Active Molecules from Snake Venoms by Snake Venomics

One of the major barriers in exploring snake venom is the low amount isolated from the venom glands, especially of rare snakes. The snake venomics will increase the discovery of the snake venom proteins and peptides for the development of new drugs for potential use [60].

### 4.2. New Drug Delivery System/Coupled with Monoclonal Antibody

One of the plausible strategies to develop clinical anti-cancer versions of cytotoxins is to conjugate the drugs with monoclonal antibodies that recognize and bind to specific epitopes on malignant cancer cells. As an example, Zhao et al. used an anti-nasopharyngeal carcinoma monoclonal antibody BAC5 conjugated with the venom of the Chinese cobra, which showed strong effects against nasopharyngeal carcinoma cells in vitro [61].

The other method for targeted cancer therapy by cytotoxin from snake venom is to combine the snake venom with silica nanoparticles. Al-Sadoon et al. demonstrated that the snake venom extracted from *Walterinnesia aegyptia* (WEV) combined with silica nanoparticles (NP) can inhibit the proliferation of human breast carcinoma cell lines and strongly induced apoptosis without significant effects on normal breast epithelial MCF-10A cells [62]. Al-Sadoon et al. also evaluated the effects of WEV+NP in the therapy of multiple myeloma in the nude mouse model. WEV+NP showed greater activities than WEV alone in decreasing the surface expression of the chemokine receptors CXCR3, CXCR4 and CXCR6 and decreased migration of the cancer cells [63], suggesting this approach possesses the promising therapeutic potential for clinical application of snake venoms.

## 5. Conclusions

In conclusion, the application of the snake venoms in cancer therapy has evolved from the usage of the crude mixtures in the 1930s into the isolation of certain biologically active components targeting specific molecular pathways. Currently, the combination of snake venoms with other technologies such as nanoparticles is still at its early stage for cancer therapy and it can be expected that more combinational treatment will emerge. Snake venoms are no doubt valuable resources for cancer drug development.

## Figures and Tables

**Table 1:** Compounds with antitumor activities isolated from snake venoms.

Target/Mechanism	Protein Names	Compounds	Snakes	Reference
antiangiogenesis	Leucurogin	disintegrin	*Bothrops leucurus*	[39]
	Contortrostatin	disintegrin	*Agkistrodon contortrix contortrix*	[40]
	Obtustatin	disintegrin	*Vipera lebetina obtusa*	[41]
	Adinbitor	disintegrin	*A. halys brevicaudus stejneger*	[42,43]
	Salmosin	disintegrin	*A. halys brevicaudus*	[44]
apoptosis induction	LAAO	LAAO	*A. halys*	[45,46]
	AHP-LAAO	LAAO	*A. halys pallas*	[47]
	LAAO	LAAO	*V. berus berus*	[48]
	disintegrin	disintegrin	*Naja naja*	[49]
	VAP and VAP2	metalloprotease/disintegrin	*Crotalus atrox*	[50,51]
	stejnitin	SVMP	*Trimeresurus stejnegeri*	[52]
